# Structural parameters associated with location of peaks of peripapillary retinal nerve fiber layer thickness in young healthy eyes

**DOI:** 10.1371/journal.pone.0177247

**Published:** 2017-05-25

**Authors:** Takehiro Yamashita, Ryo Asaoka, Yuya Kii, Hiroto Terasaki, Hiroshi Murata, Taiji Sakamoto

**Affiliations:** 1 Department of Ophthalmology, Kagoshima University Graduate School of Medical and Dental Sciences, Kagoshima, Japan; 2 Department of Ophthalmology, The University of Tokyo, Tokyo, Japan; Massachusetts Eye & Ear Infirmary, Harvard Medical School, UNITED STATES

## Abstract

The location of the peaks of the circumpapillary retinal nerve fiber layer (cpRNFL) thickness is affected by several ocular parameters. In this study, we have generated equations that can determine the peaks of the cpRNFL. This study was a prospective, observational, cross sectional study of 118 healthy right eyes. The axial length, optic disc tilt, superiortemporal (ST)- and inferiortemporal (IT)-peaks of the cpRNFL thickness, and angles of the ST and IT retinal arteries (RA) and veins (RV) were determined. The correlations between the location of the ST- and IT-peaks and ocular structural parameters and the sex, body height and weight were calculated. The best fit equations to generate the location of the ST/IT-peaks were determined using corrected-Akaike Information Criteria. The location of the ST-peak was 0.72+(0.40 x ST-RA)+(0.27 x ST-RV)+(0.14 x height)–(0.47 x papillo-macular-position)–(0.11 x disc tilt) with a coefficient of correlation of 0.61 (*P*<0.0001). The location of the IT-peak was 21.88+(0.53 x IT-RA)+(0.15 x IT-RV)+(0.041 x corneal thickness)-(1.00 x axial length) with a coefficient of correlation of 0.59 (*P*<0.0001). The location of ST/IT peaks is determined by different parameters of the ocular structure. These equations allow clinicians to obtain an accurate location of the peaks for a more accurate diagnosis of glaucoma.

## Introduction

The axons of the retinal ganglion cells (RGCs) can be damaged in eyes with increased intraocular pressure, i.e., glaucomatous eyes, and the damage is irreversible. Thus, an early diagnosis of glaucoma is essential in preventing this damage. The results of earlier studies suggested that the structural changes around the optic nerve head (ONH) [1]. and the thickness of the retinal nerve fiber layer (RNFL) around the optic disc [2] preceded the visual field (VF) defects. Thus, assessment of these parameters in eyes suspected of having glaucoma is important for an early diagnosis of glaucoma.

The improvements of optical coherence tomography (OCT) have made it possible to measure the circumpapillary RNFL (cpRNFL) thicknesses easier and more accurately [3–8]. These measurements have shown that there are two major peaks in the cpRNFL thickness profile, the supratemporal peak (ST) and the infratemporal (IT) peak. Importantly, several studies have shown that the location and height, i.e., the thickness, of the peaks of the cpRNFL can be helpful in diagnosing glaucoma [9–12].

However, there is a problem in using these parameters in healthy myopic eyes because the location and height of the peaks can be different from that of healthy emmetropic eyes [9–13]. To resolve this problem, efforts have been made to determine the profile of the cpRNFL thicknesses in healthy myopic eyes, however the findings have not been transferred to the clinic because of the many factors that can affect the cpRNFL thickness. Thus, it has been shown that the cpRNFL profile can be affected by the location of the supratemporal (ST) and infratemporal (IT) retinal arteries [9, 14–16]. In addition, the locations of the papillo-macular bundle and the ovality of the optic disc can also affect the cpRNFL thickness profile [17, 18]. Unexpectedly, it was shown that the thickness of the central and temporal regions of the retina and the axial length of the eye were significantly correlated with the body height [19–22]. In addition, we have shown that the degree of optic disc tilt can also affect the cpRNFL thickness profile [23]. Because of the many structural factors of the eye and body that can affect the cpRNFL thickness, it would be helpful to have an equation that can be used to determine the locations and heights of the peaks of the cpRNFL thickness.

The hypothesis of the current study is that the locations of cpRNFL peak angles can be better predicted by using multiple parameters, such as the locations of the retinal artery and vein, optic disc tilt and body height. Thus, the purpose of this study was to determine whether we can construct a mathematical expression or equation that can determine the location of the peaks of the cpRNFL in young healthy eyes which can then be used as the normative values to differentiate non-glaucomatous eyes from glaucomatous eyes more accurately and easily. To do this, the relationship between the locations of the peaks of the cpRNFL thickness, and the locations of the retinal arteries and veins, optic disc tilt and ovality ratio, corneal thickness, body height and weight, sex, location of the papillo-macular bundle, and the axial length were examined. The upper and lower halves of the cpRNFL were analyzed separately because the location of the damaged RNFL differs in eyes with glaucoma [24]. As a result, different parameters were identified to be related to the locations of upper and inferior peaks of cpRNFL thickness.

## Methods

### Ethics statement

All of the procedures used conformed to the tenets of the Declaration of Helsinki. A signed informed consent was obtained from all of the subjects after an explanation of the procedures to be used. The study was approved by the Ethics Committee of Kagoshima University Hospital, and it was registered with the University Hospital Medical Network (UMIN)-clinical trials registry: “Morphological analysis of the optic disc and the retinal nerve fiber in myopic eyes” (registration number was UMIN000006040). A detailed protocol is available at https://upload.umin.ac.jp/cgi-open-bin/ctr/ctr.cgi?function=brows&action=brows&type=summary&recptno=R000007154&language=J. The results presented in this manuscript are part of the overall study [9, 14].

### Subjects

This was a cross sectional, prospective, observational study of 133 eyes of 133 volunteers who were enrolled between November 1, 2010 and February 29, 2012. The participants had no known eye diseases as determined by examining their medical charts, and the data from only the right eyes were analyzed. To eliminate the ageing and cohort effects, only young adults were studied. The inclusion criteria were: age between 20 and 40 years; no pathological findings by slit—lamp biomicroscopy, ophthalmoscopy, and OCT; best corrected visual acuity (BCVA) ≤0.1 logarithm of the minimum angle of resolution (logMAR) units; and an intraocular pressure (IOP) ≤21 mmHg measured with pneumotonometer (CT-80, Topcon, Tokyo, Japan). The exclusion criteria were: known ocular diseases such as glaucoma, staphyloma, and optic disc anomalies; systemic diseases such as hypertension and diabetes; presence of VF defects; and history of refractive or intraocular surgery. None of the eyes was excluded because of poor OCT image quality caused by poor fixation.

### Measurement of axial length, central corneal thickness, and body height and weight

The axial length was measured with the AL-2000 ultrasound instrument (TOMEY, Nagoya, Japan), and the central corneal thickness was measured with the SP-3000P (TOPCON, Tokyo, Japan). The body height and weight were measured without excess clothing and shoes.

### Locations of supratemporal (ST) and infratemporal (IT) peaks of RNFL thickness, and locations of supratemporal and infratemporal retinal arteries and veins, location of papillo-macular position (PMP), and degree of optic disc ovality and tilt

The cpRNFL thickness was measured in the 3.4 mm circle scan images of the optic nerve head obtained by the TOPCON 3D OCT-1000 MARK II. In this protocol, 1024 A-scan/circles, 16 overlapping B-scan/images, and direct B-scan observations were made. The OCT images and the color fundus photographs were recorded at the same time. The optical system and software of the OCT instrument can detect the edge of the optic disc, and the scan circle is centered automatically on the optic disc just before the OCT image is recorded. To correct for errors in the centering of the scan circle, one examiner (YK) checked offline to determine that the center of the scan circle had been located at the center of the optic disc. The center of the scan circle was used as the center of the optic disc.

**Fig 1:**
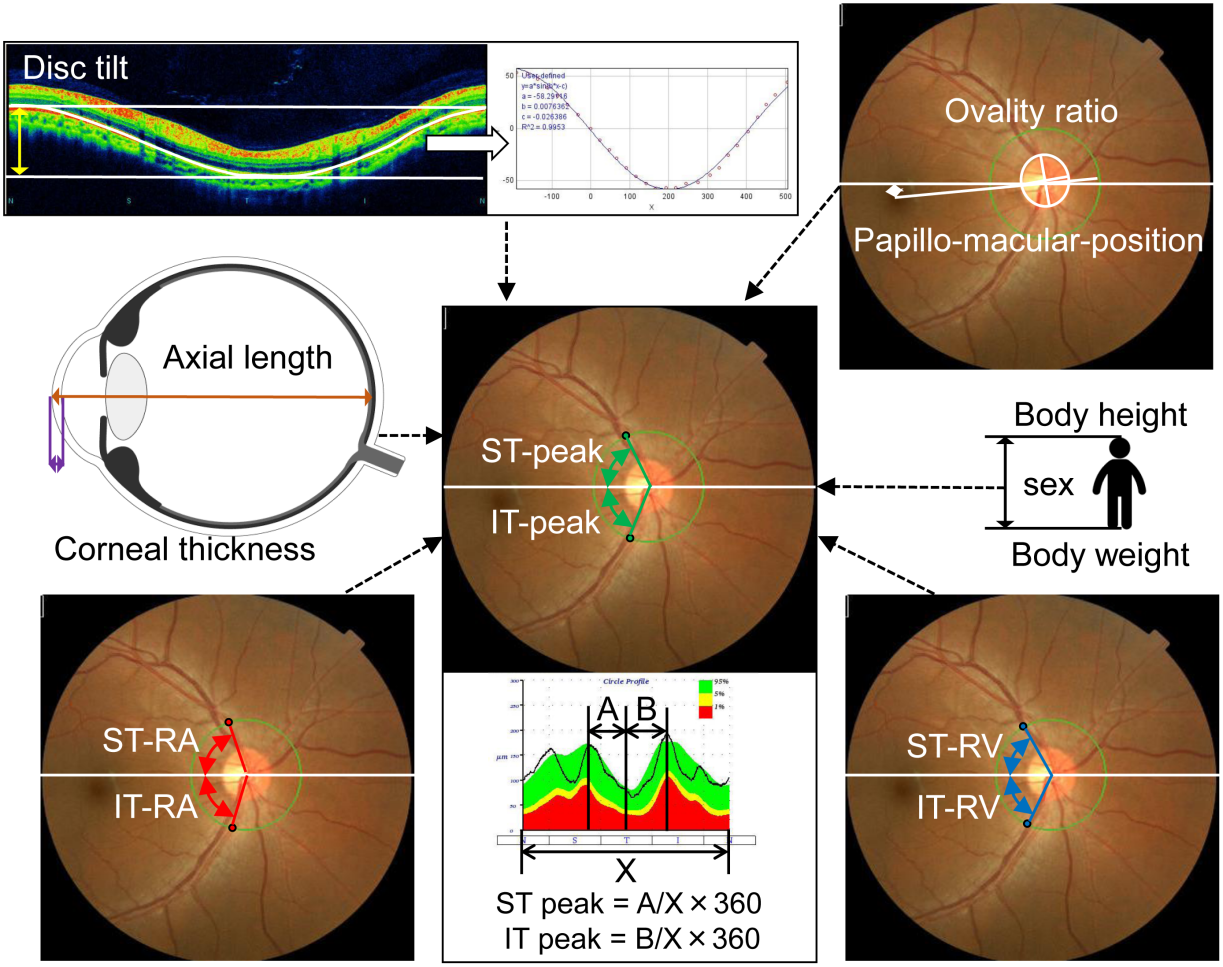
Schematic drawings of ocular and systemic parameters related to the examined ST-peak and IT-peak. ST: supratemporal, IT: infratemporal.

Then the temporal-superior-nasal-inferior-temporal (TSNIT) thickness curve was displayed and used to determine the location of the ST and IT peaks in angular values with 0° at the 12 o’clock position. The distance between the ST and IT peaks of the RNFL thickness curve was determined by dragging a vertical line in the cpRNFL thickness curve from the ST peak to the IT peak with the Photoshop CS5 program. Then, the distance between the peaks of the TSNIT curve was converted to an angular value by dividing the distance by the entire distance then multiplied by 360 (Fig 1) [9].

In the TOPCON 3D OCT-1000 MARK II, the color fundus photographs and the OCT images are taken at the same time, and a green circle was traced over the scanned circle in the photographs. Using the center of the green circle and the intersection of the ST and IT major retinal arteries and the green circle, the angle between the ST and the IT major retinal artery and the temporal horizontal line was measured. The angle between the ST or IT major retinal vein and temporal horizontal line was measured by the same method [9,10]. We named these the ST and IT retinal artery (ST- and IT-RA) and the ST and IT retinal vein (ST- and IT-RV) angles (Fig 1).

The papillo-macular position (PMP) is the angle formed by a horizontal line and the line connecting the optic disc center and the fovea in the color fundus photograph [25]. The center of the optic disc was the center of the green scan circle. The ovality ratio was determined on the color fundus photographs as described in detail [26]. The maximum and minimum disc diameters were measured by a single observer using the Photoshop software. We defined the vertically axis of the disc as the longest diameter that was less than 45 degrees of the geometric vertical axis, and the horizontal axis as the longest diameter that was more than 45 degrees of the geometric vertical axis. The degree of ovality or ovality ratio was determined by dividing the maximum by the minimum disc diameters (Fig 1).

The degree of disc tilt was determined as described in detail in our previous publication [23]. In short, the course of the retinal pigment epithelium (RPE) was marked on the B-scan images of the RNFL circle scans, and the coordinates of each pixel were determined automatically using the ImageJ program (Image J version 1.47, National Institutes of Health, Bethesda, MD; available at: http://imagej.nih.gov/ij/). The ‘x’ and ‘y’ coordinates of the B-scan images were adjusted with the center of the optic disc as the origin and the adjusted data were approximated by a sine wave equation (y = a*sin(b*x-c)) with the curve fitting program of ImageJ. The constants ‘a’, ‘b’, and ‘c’ were calculated by the least squares curve fitting program of ImageJ, and the constant ‘a’ was considered to be the degree of the optic disc tilt relative to the fovea (Fig 1). Eyes were stationary throughout the measurements and the retinal plane of the macular area was vertical to the optical axis of the eye.

### Statistical analyses

The relationships between the locations of the ST- and IT- peaks and the ST- and IT-RAs, ST- and IT-RVs, sex, body height and weight, axial length, corneal thickness, optic disc tilt, ovality ratio, and papillo-macular bundle location were determined by linear regression analysis. In this analysis, a confounding factor was that between the body height and sex because men are generally taller than women. The best linear model was then selected among all possible combinations of predictors based on the second order bias corrected Akaike Information Criterion (AICc) index. The AIC is a statistical measure used in model selection, and the A+ICc is a corrected type of the AIC, which provides an accurate estimation especially when the sample size is small [27]. It is recommended that the model selection methods be used rather than multivariate regression model to improve the model fit by removing redundant variables because the degrees of freedom in a multivariate regression model decreases with a large number of variables. The best linear models were also determined after standardizing each variable. This process does not change the variables included in the best model.

All statistical analyses were performed with the statistical programming language ‘R’ (R version 2.15.1; The Foundation for Statistical Computing, Vienna, Austria).

## Results

One hundred and thirty-three Japanese volunteers were examined. Seven eyes were excluded because of ocular diseases or prior ocular surgery, three eyes because of superior segmental optic hypoplasia, one case because of glaucoma, and three cases because of prior laser-assisted in situ keratomileusis. Eight other eyes were excluded because of difficulty in identifying the position of the peak of the RNFLT. In the end, the right eyes of 118 individuals were analyzed (Table 1).

**Table 1:** Study subjects.

Enrolled subjects	133 right eyes of 133 individuals
**Excluded subjects**	
** Superior segmental optic hypoplasia**	3 eyes
** Glaucoma**	1 eye
** Prior laser-assisted in situ keratomileusis**	3 eyes
** Difficulty in identifying the position of the peaks of the RNFLT**	8 eyes
**Analyzed subjects**	118 eyes

RNFLT: retinal nerve fiber layer thickness

**Fig 2:**
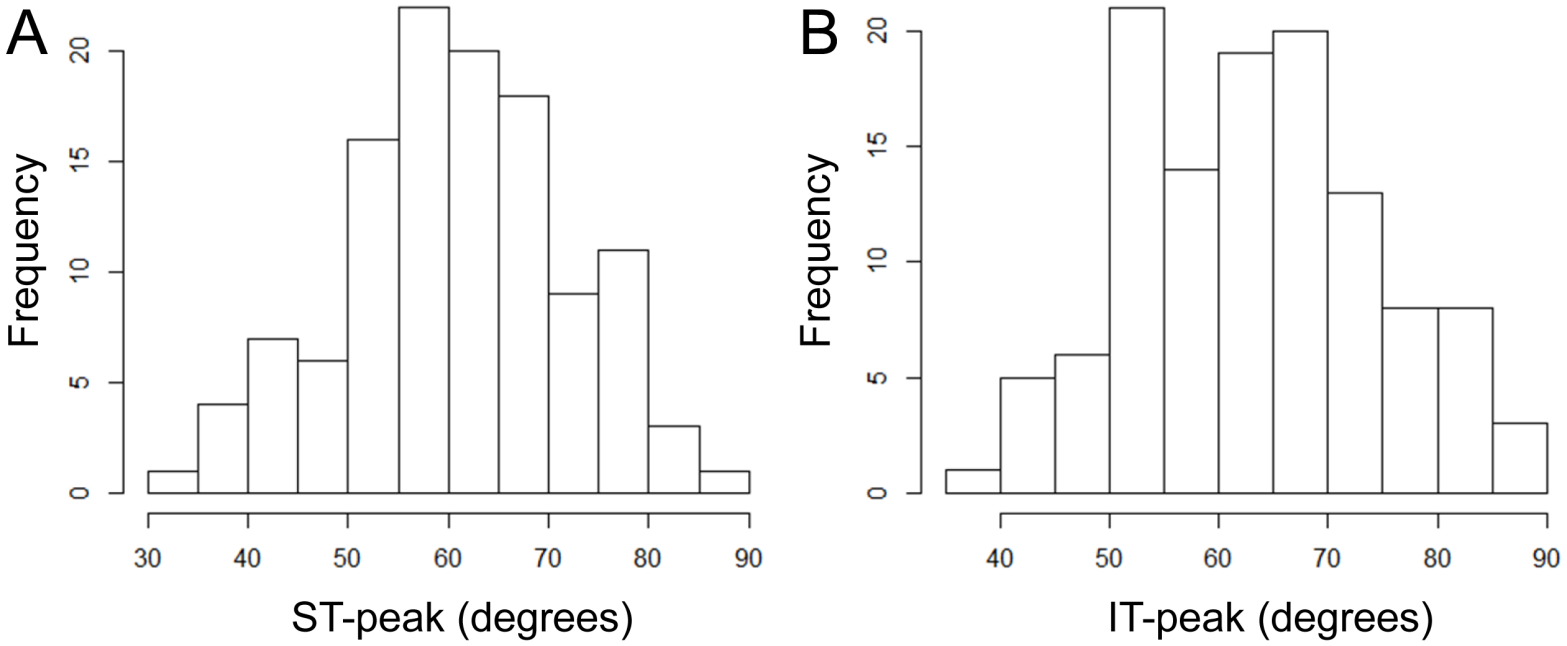
Histogram of supratemporal and infratemporal peak circumpapirally retinal nerve fiber layer thickness angle (ST- and IT-peak). ST: supratemporal, IT: infratemporal.

The demographic information of the participants is presented in Table 2. The mean ± standard deviation of the age was 25.9 ± 4.0 with a range of 22 to 40 years, and the mean axial length was 25.4 ± 1.4 with a range of 22.4 to 28.6 mm. The location of the mean ST peak was at 60.5° ±11.3° with a range of 31.2° to 86.5°, and the location of the mean IT-peak was at 63.2° ±11.7° with a range of 38.1° to 90.0° (Fig 2).

**Table 2:** The demographic information of the participants.

Demographics	Values
**Age, mean ± SD (range)**	25.9±4.0 (22 to 40)
**Sex (male: female)**	80: 38
**Height, cm, mean ± SD (range)**	167.5±8.4 (149.5 to 185.5)
**Weight, kg, mean ± SD (range)**	63.3±12.4 (42.7 to 119.0)
**Central corneal thickness, μm, mean ± SD (range)**	508.4±27.4 (435.0 to 582.0)
**Axial length, mm, mean ± SD (range)**	25.4±1.4 (22.4 to 28.6)
**Supratemporal peak cpRNFL angle, degrees, mean ± SD (range)**	60.5 ±11.3 (31.2 to 86.5)
**Infratemporal peak cpRNFL angle, degrees, mean ± SD (range)**	63.2±11.7 (38.1 to 90.0)
**Supratemporal retinal artery angle, degrees, mean ± SD (range)**	62.0 ±12.2 (29.4 to 95.2)
**Infratemporal retinal artery angle, degrees, mean ± SD (range)**	66.7 ±14.3 (24.2 to 100.4)
**Supratemporal retinal vein angle, degrees, mean ± SD (range)**	65.1 ±13.6 (27.7 to 102.2)
**Infratemporal retinal vein angle, degrees, mean ± SD (range)**	70.7 ±15.5 (39.8 to 102.1)
**Disc tilt, degrees, mean ± SD (range)**	37.6 ±17.2 (17.2 to 80.8)
**Ovality ratio, mean ± SD (range)**	0.89 ±0.11 (1.18 to 0.60)
**Pappilo-macular position, degrees, mean ± SD (range)**	5.5 ±3.4 (-3.58 to 12.8)

SD: standard deviation, cpRNFL: circumpapillary retinal nerve fibre layer

The ST-RA, ST-RV, body height, PMP, and optic disc tilt were found to be significant associated with the location of the ST-peak. The best-selected equation to determine the location of the ST peak was;

ST-peak=0.72+(0.40 x ST-RA)+(0.27 x ST-RV)+(0.14 x height)–(0.47 x PMP)–(0.11 x disc tilt).

The coefficient of correlation for this relationship was adjusted r^2^ = 0.51 (*P* <0.0001). After standardization of each variable, the equation model was;

$$\text{ST-peak}=\left(4.8\text{ x }10^{-16}\right)\;+\;(0.44\text{ x ST-RA})\;+\;\left(0.33\text{ x ST-RV}\right)\;+\;\left(0.11\text{ x height}\right)–\left(0.14\text{ x PMP}\right)–\left(0.17\text{ x disc tilt}\right).$$

In the best model for the IT-peak, IT-RA, IT-RV, corneal thickness, and axial length were selected as significant predictors. The equation for the best-selected model was;

IT-peak=21.88+(0.53 x IT-RA)+(0.15 x IT-RV)+(0.041 x corneal thickness)–(1.00 x axial length).

The coefficient of correlation was adjusted r^2^ = 0.59 (*P* <0.0001). After standardization of each variable, this equation was calculated as;

$$\text{IT-peak}=\left(-1.4\text{ x }10^{-16}\right)+(0.65\text{ x IT-RA})+\left(0.19\text{ x IT-RV}\right)+\left(0.096\text{ x corneal thickness}\right)–\left(0.12\text{ x axial length}\right).$$

## Discussion

We have determined equations that can calculate the locations (in degrees) of the ST- and the IT-peak thicknesses by using the contributions of the different factors correlated with the ST- and IT-peaks.

During the analyses, many issues were raised. First, the peak of the RNFL thickness was significantly correlated with the angles made by the ST and IT major arteries and veins with the line connecting the fovea and the center of the optic disc. The standardized index was 5.44 for the ST artery and 10.15 for the IT artery. The angle of the retinal artery was the strongest parameter in this polynomial equation. Similarly, the angle of the retinal vein was the second strongest factor; 4.68 for the ST-vein and 3.02 for the IT vein. This is consistent with our earlier findings that the trajectory of the supra- and infra-temporal retinal nerve fiber bundles were significantly correlated with the trajectory of the supra- and infra-temporal retinal arteries and veins [14].

**Fig 3:**
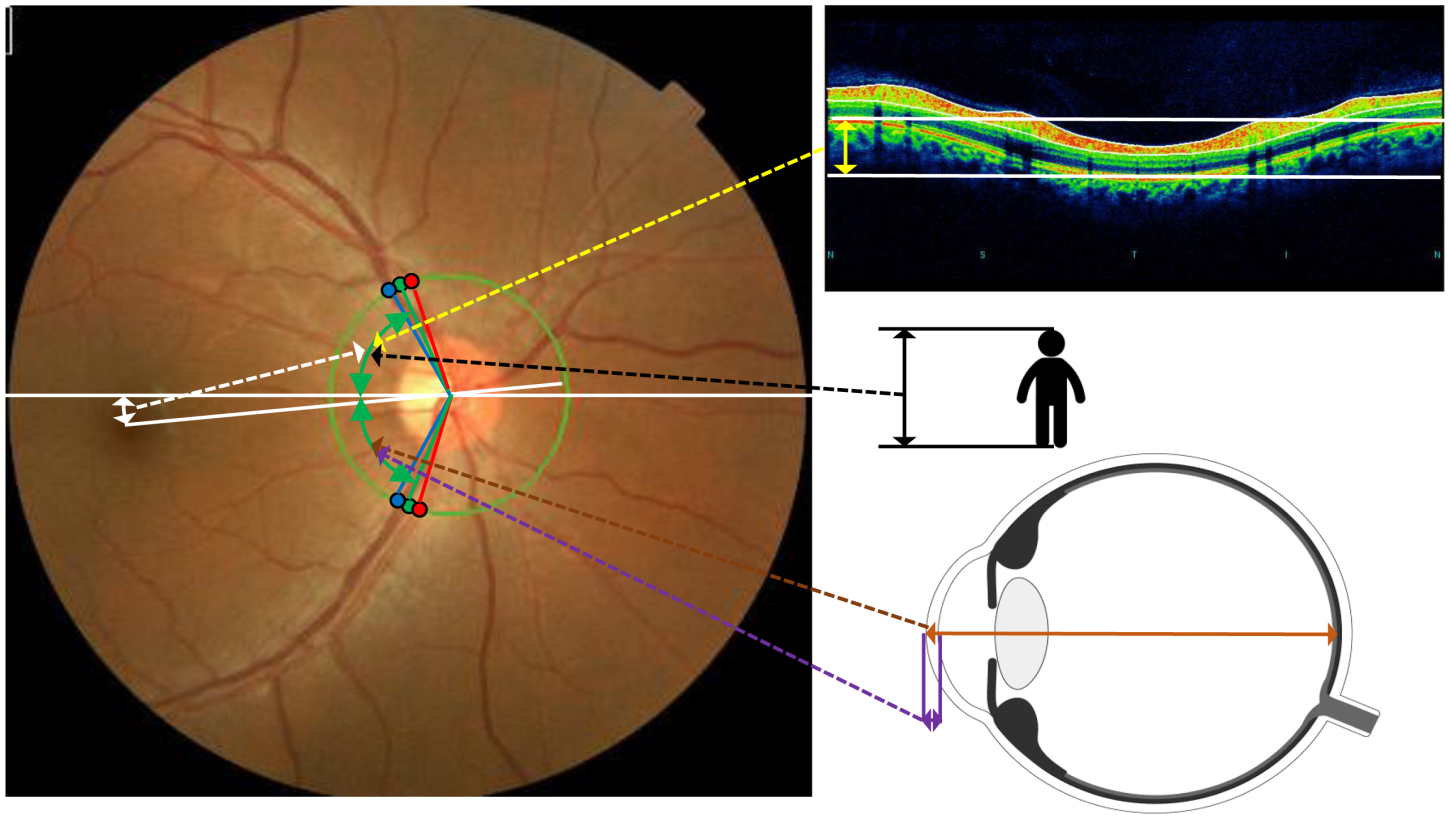
Factors affect supratemporal RNFL peak thickness position.

It is important to note that the variables selected by the statistical analyses differed for the ST peak and IT peak (Fig 2). For the ST-peak, the optic disc tilt, PMP, and body height were found to be significantly correlated to the ST peak. The more the optic disc tilted, the smaller was the thickness of the RNFL at its peak. Thus, the ST-peak was closer to the macula in an eye whose optic disc tilted more. The degree of optic disc tilt was significantly associated with the angle it made with the visual axis and the PMP. These findings indicate the relative location of the center of the optic disc relative to the fovea. Therefore, these results suggest that the ST RNFLT peak location was affected by the relative three-dimensional location of the optic disc against the fovea (Fig 3).

Supratemporal RNFL peak thickness position (upper green double arrow heads) is affected by the location of the papillo-macular position (white double arrow heads), optic disc tilt (yellow double arrow heads) and body height (black double arrow heads). Infratemporal RNFL peak position (lower green double arrow heads) was affected by corneal thickness (purple double arrow heads) and axial length (brown double arrow heads). Both RNFL peaks were affected by retinal artery position and retinal vein position (blue and red points).

There was a tendency for the location of the ST peak to be larger as the body height increased. Most population-based studies have shown a significant positive correlation between the body height and axial length of the eye [20–22]. However, there was no significant correlation between the body height and the axial length in the subjects in this study. The location of the IT peak was not significantly correlated with the body height. The enlargement of eye after birth must be more complicated than is generally believed. More detailed analyses are required to learn how the different structures of eye and body affect the RNFL thickness and the locations of the peaks. We are performing a cohort study on the relationship between growth of the body and the eye structures in a Japanese population which should provide insights on this relationship.

The location of the IT peak of the RNFL thickness was significantly correlated with the corneal thickness and axial length. Specifically, the angle of the IT peak was closer to fovea in eyes with a longer axial length and a thinner cornea. It is interesting to note that a thin cornea and long axial length are established risk factors for open angle glaucoma. In addition, the VF changes in the early stages of glaucoma are mainly observed in the lower temporal region more than the upper temporal region [24,28–31]. The present finding may explain why this relationship exists in eyes with early glaucoma.

It was somewhat unexpected to find that the structural changes associated with the RNFL thickness were different for the upper and lower retina. There must be different factors in the growth and developmental pattern in the upper and lower halves of the eye. Indeed, the initial changes of visual field defects in glaucoma are not necessarily the same in the lower and upper fields. This issue needs further investigation in glaucomatous eyes.

This study has limitations. One limitation was that the study population was made up of young Japanese volunteers who are known to belong to the most myopic group in the world [32]. Thus, our results describe the characteristics of young myopic eyes, but might not necessarily hold for older and non-myopic populations. On the other hand, the reliability of the examination was very high because no pathological factors such as cataract or vitreal opacities were present in young healthy individuals and the understanding of the examination was high. In addition, the narrow range of age prevented the interference of cohort effects and aging effects. Another limitation was that the number of female participants was fewer than male participants (80 men vs 38 women). We performed multiple regression analysis with sex, however the effect of sex could not be clearly observed in the current study. However it has been reported that there is a sex difference in the development of glaucoma [33]., and different results could be observed when the investigations were carried out collecting larger number of female participants. An epidemiological study should help generalizing the present results to other populations.

In conclusion, we determined an equation that can calculate the location of the peak of the cpRNFL thickness. The angles of the retinal arteries and veins were strongly correlated with both the ST- and IT-peaks. The body height, papillo-macular location, and optic disc tilt were significantly correlated to the position of ST-peak. The corneal thickness and axial length were significantly correlated with the location of the IT-peak. This information should be useful in determining the pathological mechanism causing the RNFL damage of either the upper or lower retinal areas in glaucomatous eyes.

## Supporting information

S1 Raw Data (CSV)

## References

[1] Quigley HA, Katz J, Derick RJ, Gilbert D, Sommer A. An evaluation of optic disc and nerve fiber layer examinations in monitoring progression of early glaucoma damage. Ophthalmology 1992;99:19–28 1741133 10.1016/s0161-6420(92)32018-4

[2] Sommer A, Katz J, Quigley HA, Miller NR, Robin AL, Richter RC, et al Clinically detectable nerve fiber atrophy precedes the onset of glaucomatous field loss. Arch Ophthalmol 1991;109:77–83 1987954 10.1001/archopht.1991.01080010079037

[3] Blumenthal EZ, Williams JM, Weinreb RN, Girkin CA, Berry CC, Linda M, Zangwill LM. Reproducibility of nerve fiber layer thickness measurements by use of optical coherence tomography. Ophthalmology. 2000;107:2278–82. 11097610 10.1016/s0161-6420(00)00341-9

[4] Huang D, Swanson EA, Lin CP, Schuman JS, Stinson WG, Chang W, et al Optical coherence tomography. Science. 1991;254:1178–81. 1957169 10.1126/science.1957169 PMC4638169

[5] Mistlberger A, Liebmann JM, Greenfield DS, Pons ME, Hoh ST, Ishikawa H, et al Heidelberg retina tomography and optical coherence tomography in normal, ocular-hypertensive, and glaucomatous eyes. Ophthalmology. 1999;106:2027–32. 10.1016/S0161-6420(99)90419-0 10519603

[6] Zangwill LM, Williams J, Berry CC, Knauer S, Weinreb RN. A comparison of optical coherence tomography and retinal nerve fiber layer photography for detection of nerve fiber layer damage in glaucoma. Ophthalmology. 2000;107:1309–15. 10889104 10.1016/s0161-6420(00)00168-8

[7] Leung CK, Mohamed S, Leung KS, Cheung CYL, Chan SL, Cheng DK, et al Retinal Nerve Fiber Layer Measurements in Myopia: An Optical Coherence Tomography Study. Invest Ophthalmol Vis Sci. 2006;47:5171–6. 10.1167/iovs.06-0545 17122099

[8] Hoh ST, Lim MC, Seah SK, Lim AT, Chew SJ, Foster PJ, et al Peripapillary Retinal Nerve Fiber Layer Thickness Variations with Myopia. Ophthalmology. 2006;113:773–7. 10.1016/j.ophtha.2006.01.058 16650672

[9] Yamashita T, Asaoka R, Tanaka M, Kii Y, Yamashita T, Nakao K, et al Relationship between position of peak retinal nerve fiber layer thickness and retinal arteries on sectoral retinal nerve fiber layer thickness. Invest Ophthalmol Vis Sci. 2013;54:5481–8. 10.1167/iovs.12-11008 23847316

[10] Yoo YC, Lee CM, Park JH. Changes in peripapillary retinal nerve fiber layer distribution by axial length. Optom Vis Sci. 2012;89:4–11. 10.1097/OPX.0b013e3182358008 21983121

[11] Hong SW, Ahn MD, Kang SH, Im SK. Analysis of peripapillary retinal nerve fiber distribution in normal young adults. Invest Ophthalmol Vis Sci. 2010;51:3515–23. 10.1167/iovs.09-4888 20164448

[12] Yamashita T, Kii Y, Tanaka M, Yoshinaga W, Yamashita T, Nakao K, et al Relationship between supernormal sectors of retinal nerve fibre layer and axial length in normal eyes. Acta Ophthalmol. 2014;92:e481–7. 10.1111/aos.12382 24655430

[13] Fujino Y, Yamashita T, Murata H, Asaoka R. Adjusting Circumpapillary Retinal Nerve Fiber Layer Profile Using Retinal Artery Position Improves the Structure-Function Relationship in Glaucoma. Invest Ophthalmol Vis Sci. 2016;57:3152–8. 10.1167/iovs.16-19461 27309619

[14] Yamashita T, Sakamoto T, Terasaki H, Tanaka M, Kii Y, Nakao K. Quantification of retinal nerve fiber and retinal artery trajectories using second-order polynomial equation and its association with axial length. Invest Ophthalmol Vis Sci. 2014;55:5176–82. 10.1167/iovs.14-14105 25074777

[15] Hood DC, Fortune B, Arthur SN, Xing D, Salant JA, Ritch R, et al Blood vessel contributions to retinal nerve fiber layer thickness profiles measured with optical coherence tomography. J Glaucoma. 2008;17:519–28. 10.1097/IJG.0b013e3181629a02 18854727 PMC2987575

[16] Hood DC, Salant JA, Arthur SN, Ritch R, Liebmann JM. The location of the inferior and superior temporal blood vessels and interindividual variability of the retinal nerve fiber layer thickness. J Glaucoma. 2010;19:158–66. 10.1097/IJG.0b013e3181af31ec 19661824 PMC2889235

[17] Lamparter J, Russell RA, Zhu H, Asaoka R, Yamashita T, Ho T, et al The influence of inter-subject variability in ocular anatomical variables on the mapping of retinal locations to the retinal nerve fiber layer and optic nerve head. Invest Ophthalmol Vis Sci. 2013;54: 6074–82. 10.1167/iovs.13-11902 23882689

[18] Hong S, Kim CY, Seong GJ. Adjusted peripapillary retinal nerve fiber layer thickness measurements based on the optic nerve head scan angle. Invest Ophthalmol Vis Sci. 2010;51:4067–74. 10.1167/iovs.09-4301 20237251

[19] Yamashita T, Tanaka M, Kii Y, Nakao K, Sakamoto T. Association between retinal thickness of 64 sectors in posterior pole determined by optical coherence tomography and axial length and body height. Invest Ophthalmol Vis Sci. 2013;54:7478–82. 10.1167/iovs.13-12586 24168996

[20] Yin G, Wang YX, Zheng ZY, Yang H, Xu L, Jonas JB, et al Ocular axial length and its associations in Chinese: the Beijing Eye Study. PLoS One. 2012;7:e43172 10.1371/journal.pone.0043172 22927949 PMC3424225

[21] Wang D, Ding X, Liu B, Zhang J, He M. Longitudinal changes of axial length and height are associated and concomitant in children. Invest Ophthalmol Vis Sci. 2011;52:7949–53. 10.1167/iovs.11-7684 21896861

[22] Nangia V, Jonas JB, Matin A, Kulkarni M, Sinha A, Gupta R. Body height and ocular dimensions in the adult population in rural Central India. The Central India Eye and Medical Study. Graefes Arch Clin Exp Ophthalmol. 2010;248:1657–66. 10.1007/s00417-010-1448-0 20652306

[23] Yamashita T, Sakamoto T, Yoshihara N, Terasaki H, Kii Y, Tanaka M, et al Circumpapillary course of retinal pigment epithelium can be fit to sine wave and amplitude of sine wave is significantly correlated with ovality ratio of optic disc. PLoS One. 2015;10:e0122191 10.1371/journal.pone.0122191 25848777 PMC4388545

[24] Motolko M, Drance SM, Douglas GR. Visual field defects in low-tension glaucoma. Comparison of defects in low-tension glaucoma and chronic open angle glaucoma. Arch Ophthalmol. 1982;100:1074–7. 7092645 10.1001/archopht.1982.01030040052005

[25] Garway-Heath DF, Poinoosawmy D, Fitzke FW, Hitchings RA. Mapping the visual field to the optic disc in normal tension glaucoma eyes. Ophthalmology. 2000;100:1809–15. 10.1016/s0161-6420(00)00284-0 11013178

[26] Tay E, Seah SK, Chan SP, Lim AT, Chew SJ, Foster PJ, et al Optic disk ovality as an index of tilt and its relationship to myopia and perimetry. Am J Ophthalmol. 2005;139: 247–52. 10.1016/j.ajo.2004.08.076 15733984

[27] Burnham KP, Anderson DR. Multimodel Inference: Understanding AIC and BIC in Model Selection., Sociological Methods & Research 2004, 33:261–304

[28] Gordon MO, Beiser JA, Brandt JD, Heuer DK, Higginbotham EJ, Johnson CA, et al The Ocular Hypertension Treatment Study: baseline factors that predict the onset of primary open-angle glaucoma. Arch Ophthalmol. 2002;120:714–20 12049575 10.1001/archopht.120.6.714

[29] Leung DY, Tham CC, Li FC, Kwong YY, Chi SC, Lam DS. Silent cerebral infarct and visual field progression in newly diagnosed normal-tension glaucoma: a cohort study. Ophthalmology. 2009;116:1250–6. 10.1016/j.ophtha.2009.02.003 19481813

[30] Hollands H, Johnson D, Hollands S, Simel DL, Jinapriya D, Sharma S. Do findings on routine examination identify patients at risk for primary open-angle glaucoma? The rational clinical examination systematic review. JAMA. 2013;309:2035–42. 10.1001/jama.2013.5099 23677315

[31] Suzuki Y, Iwase A, Araie M, Yamamoto T, Abe H, Shirato S, et al Risk factors for open-angle glaucoma in a Japanese population: the Tajimi Study. Ophthalmology. 2006;113:1613–7. 10.1016/j.ophtha.2006.03.059 16828504

[32] Sawada A, Tomidokoro A, Araie M, Iwase A, Yamamoto T; Tajimi Study Group. Refractive errors in an elderly Japanese population: the Tajimi study. Ophthalmology. 2008;115:363–70. 10.1016/j.ophtha.2007.03.075 18243904

[33] Vajaranant TS, Nayak S, Wilensky JT, Joslin CE. Gender and glaucoma: what we know and what we need to know. Curr Opin Ophthalmol. 2010; 21:91–9. 10.1097/ICU.0b013e3283360b7e 20051857 PMC4326058

